# Association between ePWV and delirium risk in icu sepsis patients: a retrospective analysis based on the MIMIC database and development of a predictive model

**DOI:** 10.1016/j.clinsp.2026.101106

**Published:** 2026-09-05

**Authors:** Lihong Dong, Xixiang Yang, Xiaolin Zhu, Hong Wang, Xiaoyan Wang

**Affiliations:** a Department of Critical Care Medicine, The First Affiliated Hospital of Kunming Medical University, Kunming Yunnan, China; b Ningxia Medical University, Yinchuan Ningxia, China; c Department of Critical Care Medicine, The Sixth Affiliated Hospital of Kunming Medical University, Yuxi Yunnan, China

**Keywords:** Sepsis, Delirium, ePWV, Machine learning, MIMIC-IV

## Abstract

**Background:**

Sepsis, frequently complicated by delirium with poor prognosis, is common in the Intensive Care Unit (ICU). Estimated Pulse Wave Velocity (ePWV), a non-invasive arterial stiffness marker, remains unexplored regarding delirium risk in ICU sepsis patients. This study investigated ePWV's association with sepsis-linked delirium and developed a predictive model.

**Methods:**

This retrospective cohort study utilized MIMIC-IV 3.1 data. ICU patients with sepsis meeting the Sepsis-3.0 criteria were randomly allocated in a 7:3 to the training and validation cohorts.The exposure was ePWV derived using age and blood pressure. The outcome was delirium, defined by the Confusion Assessment Method for the Intensive Care Unit (CAM-ICU). Features were selected via Boruta and LASSO.Associations were examined using Cox models, Kaplan-Meier curves, restricted cubic splines, and subgroup analyses. Five machine learning models were evaluated.

**Results:**

Among 24,889 patients (delirium incidence 44.4%), Kaplan-Meier analysis showed significant differences in delirium risk across ePWV levels (p < 0.0001). Cox regression indicated ePWV was positively associated with delirium (HR = 1.018, 95% CI: 1.008‒1.027), with the highest quartile showing 19% elevated risk versus the lowest (HR = 1.193). RCS analysis revealed a nonlinear relationship (p = 0.019). Subgroup analyses were significant except for cerebral infarction. K-Nearest Neighbor performed best with AUC = 0.736 in validation. SHAP analysis demonstrated respiratory failure, ARFand ACEI as key features.

**Conclusion:**

ePWV was independently associated with delirium risk in ICU sepsis patients. The machine learning model demonstrated good discriminative ability, facilitating early identification of high-risk patients.

## Highlights

• ePWV is an independent predictor of delirium in ICU sepsis patients.
• Nonlinear ePWV-delirium relationship shown via RCS analysis.
• KNN model with ePWV achieves AUC = 0.736 in validation cohort.
• SHAP identifies RF, ARF, and ACEI as key predictors.
• ePWV is a novel non-invasive biomarker for early delirium screening.

## Introduction

Sepsis is a leading fatal condition in the Intensive Care Unit (ICU). It fundamentally features life-threatening organ dysfunction owing to a dysregulated host response to infection, with high incidence and death rates.^1^ Globally, approximately 48 million individuals develop sepsis each year, resulting in about 11 million deaths.^2^ During the complex pathophysiological process of sepsis, involvement of the central nervous system is highly prevalent and often manifests as acute brain dysfunction, namely delirium. Its clinical manifestations encompass altered consciousness, attention deficits, disorientation, hallucinations, and delusions.^3^ Its incidence among ICU sepsis patients remains high, reported to range from 17.7% to 48%. Its severity is closely linked to elevated mortality, longer hospital stay, long-term cognitive damage, and greater healthcare burden.^4^^,^^5^ These findings indicated that early high-risk patient identification and timely interventions are crucial for ameliorating the prognosis of sepsis patients.

Arterial Stiffness (AS), a well-established marker of vascular health, has demonstrated prognostic value across varied diseases. Increased AS reflects endothelial injury and vascular dysfunction.^6^^,^^7^ Atherosclerosis, associated with increased AS and commonly regarded as a marker of vascular aging and systemic damage, is independently associated with cardiovascular diseases.^8^ Beyond its relation to cardiovascular and cerebrovascular events, AS may also be associated with sepsis-related encephalopathy development and progression by impairing cerebral autoregulation, exacerbating microcirculatory dysfunction, and promoting systemic inflammation.^9^^,^^10^ Estimated Pulse Wave Velocity (ePWV), a new, non-invasive vascular function indicator, is derived based on age and Blood Pressure (BP). It estimates aortic stiffness through a formula and reflects the elasticity and stiffness of large arteries without the need for complex equipment.^11^ ePWV is related to the prognosis of various diseases, including coronary heart disease,^12^^,^^13^ subarachnoid hemorrhage,^14^ Chronic Kidney Disease (CKD),^15^ and atherosclerotic heart disease,^15^ indicating its broad applicability as a vascular biomarker.

However, the specific association between ePWV and the delirium risk in ICU sepsis patients remains unexplored. This retrospective cohort study, utilizing the large public database MIMIC-IV, aimed at unraveling the relation of ePWV to delirium risk in ICU sepsis patients. Furthermore, our study sought to lay the groundwork for timely intervention and improved outcomes via early, non-invasive high-risk patient identification by constructing and corroborating a clinical predictive model incorporating ePWV.

## Methods

### Study population

Our study leveraged MIMIC-IV 3.1, which contains 2008‒2022 information on patients hospitalized at the Beth Israel Deaconess Medical Center (BIDMC).^16^ To protect patient privacy, personal information was de-identified and replaced with randomly generated identifiers, so ethical approval and informed consent were waived. The first author (LhD) completed the Collaborative Institutional Training Initiative (CITI) program and passed the “Conflicts of Interest” and “Data or Specimens Only Research” examinations (ID: 14,326,940), thereby obtaining authorization to access and extract required variables. This study followed the STROBE Statement guidelines.^17^

ICU sepsis patients were analyzed. Sepsis was confirmed per Sepsis-3.0 as a SOFA score ≥ 2 in the setting of confirmed or suspected (antibiotic administration within 24 h or within 3-days after culture sampling) infection. Exclusion criteria were: 1) ICU stay < 12 h; 2) Age < 18; 3) Missing BP data (Systolic Blood Pressure [SBP] and Diastolic Blood Pressure [DBP]); 4) For patients with recurrent ICU entries for sepsis, only the first admission was analyzed.

### Data collection

MIMIC-IV data were extracted via PostgreSQL 18 and Navicat Premium 17.2.3 through SQL and encompassed: 1) Demographic information: age, sex, and ethnicity; 2) Vital signs: SBP, DBP, Mean Blood Pressure (MBP), Oxygen Saturation (SpO_2_), Respiratory Rate (RR), and heart rate; 3) Comorbidities identified per ICD-9 or ICD-10 codes: Acute Renal Failure (ARF), atrial fibrillation, cardiac failure, Cerebral Hemorrhage (CH), cerebral infarction, CKD, Chronic Obstructive Pulmonary Disease (COPD), dementia, depression, diabetes, Hepatic Failure (HF), hypertension, hypovolemia, Ischemic Heart Disease (IHD), liver cirrhosis, malignant cancer, Respiratory Failure (RF); 4) Laboratory parameters: Activated Partial Thromboplastin Time (APTT), blood glucose, creatinine, Hematocrit (HCT), Hemoglobin (HGB), International Normalized Ratio (INR), lactate, Platelet Count (PLT), Prothrombin Time (PT), urea nitrogen, White Blood Cell (WBC); 5) ICU scoring systems: APSIII, GCS, Logistic Organ Dysfunction System (LODS), Sequential Organ Failure Assessment (SOFA), Systemic Inflammatory Response Syndrome (SIRS); 6) Medications: Angiotensin-Converting Enzyme Inhibitor (ACEI), antibiotics, vasoactive drugs, and sedative agents. To minimize reverse causality as much as possible, the extraction of vital signs, laboratory indicators, and medication treatment data was restricted to the period before the onset of delirium. For variables with multiple measurements, the first recorded value within 72 h after ICU entry was extracted.

As for the handling of missing data, to minimize selection bias caused by case deletion, multiple imputation based on Random Forest (RF) was applied for variables with <20% missing data. For variables with >20% missingness, missing values were categorized and included in the analysis as dummy variables. Notably, lactate had a missing rate exceeding 20%.

### Exposure and outcome

The primary exposure was ePWV derived from age and BP. MBP was DBP + 0.4 × (SBP - DBP).^18^ Subsequently, ePWV was^19^ 9.587 - (0.402 × age) + (4.560 × 10^–3^ × age^2^) - (2.621 × 10^–5^ × age^2^ × MBP) + (3.176 × 10^–3^ × age × MBP) - (1.832 × 10^–2^ × MBP). Outliers were addressed by excluding values below the 1st percentile and above the 99th percentile. Patients were split into Q1‒Q4 based on ePWV to further examine the relation of ePWV level to outcomes.

The outcome was defined as the risk of first-onset delirium in ICU patients with sepsis. Delirium was primarily assessed using the widely applied clinical tool, the Confusion Assessment Method for the intensive care unit (CAM-ICU).^20^ A diagnosis of delirium required a RASS score ≥ −3 with acute onset or fluctuating mental status (Feature 1), inattention (Feature 2), and disorganized thinking (Feature 3) or altered consciousness level (Feature 4). Values and measurement times of the Richmond Agitation-Sedation Scale (RASS) score and the four CAM-ICU features were obtained from the ICU chartevents module.

### Statistical analysis

Normality of continuous variables was assessed employing the Kolmogorov-Smirnov test. Normally distributed ones were shown in mean ± Standard Deviation (SD) and compared with Student’s *t*‑test or ANOVA, while non‑normally distributed ones were in median (Interquartile Range, IQR) and compared via the Mann-Whitney *U* or Kruskal-Wallis H test. Categorical variables were shown in percentages (%) and compared employing the Pearson χ² or Fisher’s exact test.

Kaplan-Meier (KM) curves presented differences in cumulative event incidence among patients with different ePWV levels.

According to the principle of random allocation, the dataset was split into training and validation cohorts at a 7:3 ratio, with the former for feature selection and model development and the latter for performance evaluation. Features were selected via the Boruta algorithm and LASSO regression. Boruta is an RF-based feature selection algorithm whose core concept is to systematically evaluate the importance of all original features by constructing shadow features. Specifically, it compares original features with randomly permuted shadow features and ultimately classifies variables into “important”, “rejected”, or “tentative”, thereby identifying features related to the outcome.^21^ LASSO regression is a linear regression approach with L1 regularization that penalizes coefficients, shrinking those of less important variables toward zero and achieving automatic feature selection while improving model simplicity and stability.^22^ The intersection of features identified via both approaches was selected to determine the ultimate set of important variables and reduce redundancy.

The relation of ePWV to delirium in ICU sepsis population was explored via Cox proportional hazards models, with stepwise confounder adjustment: Model 1: crude; Model 2: involved adjustment of age, sex, and ethnicity; Model 3: additionally adjusted for variables selected via Boruta and LASSO. The possible nonlinear relation of ePWV to delirium risk was examined in Restricted Cubic Spline (RCS) analysis. The consistency of relations across populations was assessed employing subgroup analyses. Based on variables from Model 3 and the MIMIC population, five Machine Learning (ML) models were developed: RF, Neural Network Model (NNM), Gradient Boosted Model (GBM), Naïve Bayes (NB), and K-Nearest Neighbor (KNN). Hyperparameters were optimized using random search with 100 iterations. Model performance was rated employing Receiver Operating Characteristic (ROC), decision, and calibration curves, along with sensitivity, specificity, balanced accuracy, and F1 score. The optimal model was identified by model performance comparison using Integrated Discrimination Improvement (IDI) and Net Reclassification Improvement (NRI). Finally, Model interpretability was enhanced by employing SHAP analysis. Data were processed, analyzed, and visualized via R 4.4.3, and p < 0.05 signified statistical significance.

### Ethics statement

This study used de-identified data from the MIMIC‑IV database, which was ethically approved by the ethics committee of BIDMC, waiving the need for individual informed consent. The first author (LhD) obtained data access after completing the CITI program (ID: 14,326,940) and signing a data use agreement. All analyses complied with applicable ethical standards.

## Results

### Baseline characteristics

**Fig. 1:**
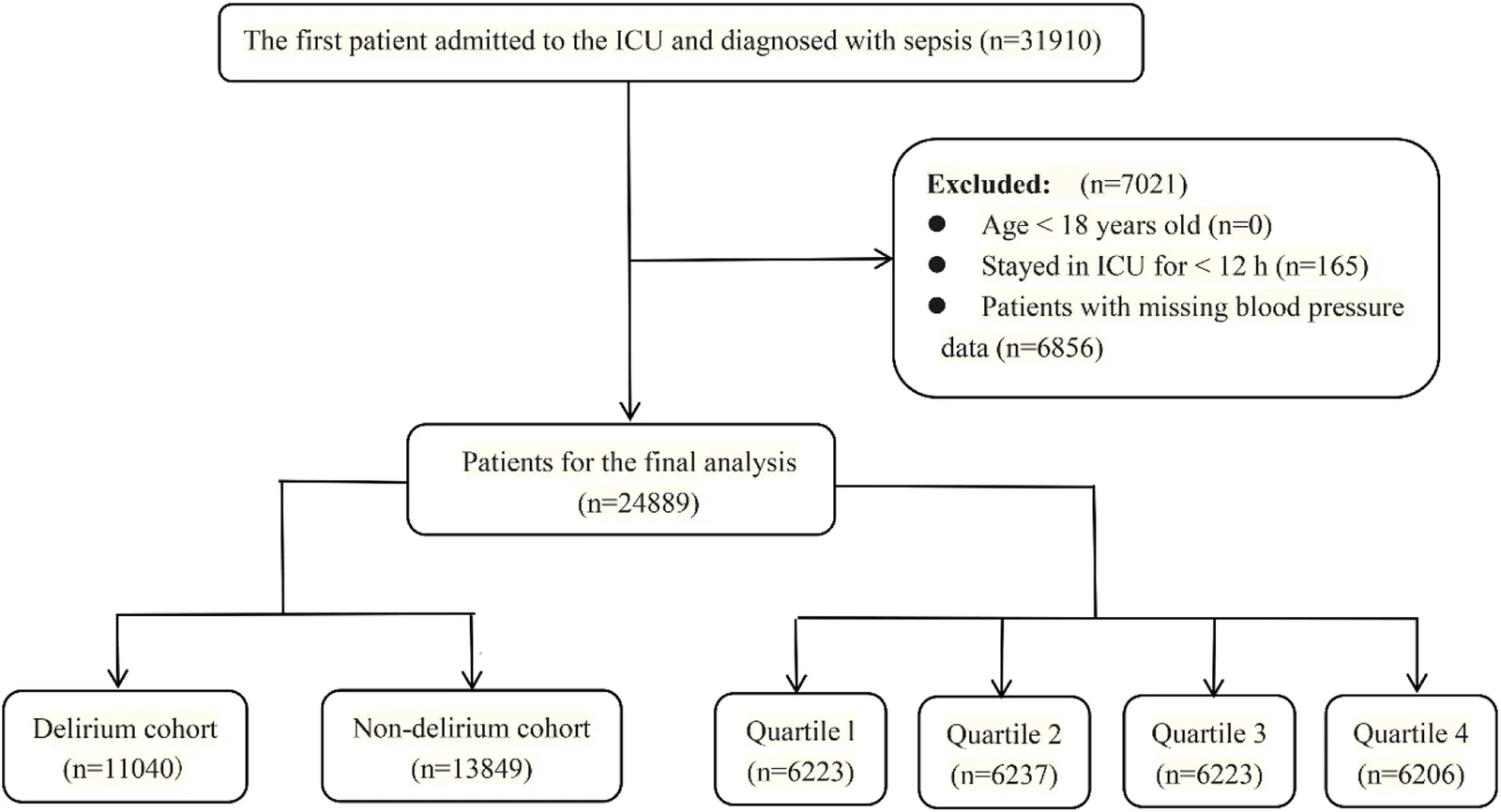
Flowchart for participant selection.

24,889 patients were encompassed per the eligibility criteria. The selection process is provided in Fig. 1.

Table 1 summarizes baseline characteristics by delirium status. Among the total cohort, 11,040 patients (44.4%) developed delirium, while 13,849 patients (55.6%) did not. The median age was 68, with more men (58%) and a predominance of White patients (66%). The delirium cohort was notably older and stayed longer in the hospital. They also had higher risks of comorbidities, including ARF, cardiac failure, CH, CKD, COPD, diabetes, HF, hypovolemia, and RF, whereas the proportions of hypertension and IHD were lower (p < 0.001, p = 0.008). Disease severity scores (e.g., APS III and SOFA) were markedly higher in the delirium cohort (both p < 0.001). Moreover, ePWV was notably higher in delirium patients relative to those without delirium (p < 0.001), indicating more severe AS. Laboratory parameters like creatinine, glucose, lactate, and WBC were also elevated in the delirium group.

**Table 1:** Baseline characteristics of the study population based on the grouping of whether delirium occurred.

Characteristic	Overall (n = 24,889)^a^	Non-delirium (n = 13,849)^a^	Delirium (n = 11,040)^a^	p-value^b^^,^^c^
Follow-up time (delirium)(days)	4.0 [1.1, 7.4]	6.3 [4.3, 10.1]	1.0 [0.3, 2.4]	<0.001⁎⁎⁎
Age (years)	68 [57, 79]	68 [57, 79]	69 [58, 80]	<0.001⁎⁎⁎
Gender (%)				0.053
Female	10,531 (42)	5785 (42)	4746 (43)	
Male	14,358 (58)	8064 (58)	6294 (57)	
Race (%)				<0.001⁎⁎⁎
Asian	744 (3.0)	448 (3.2)	296 (2.7)	
Black	2326 (9.3)	1202 (8.7)	1124 (10)	
Hispanic	857 (3.4)	496 (3.6)	361 (3.3)	
Other	4636 (19)	2053 (15)	2583 (23)	
White	16,326 (66)	9650 (70)	6676 (60)	
ARF (%)	10,320 (41)	4801 (35)	5519 (50)	<0.001⁎⁎⁎
Atrial fibrillation (%)	5779 (23)	3470 (25)	2309 (21)	<0.001⁎⁎⁎
Cardiac failure (%)	7149 (29)	3617 (26)	3532 (32)	<0.001⁎⁎⁎
Cerebral hemorrhage (%)	1482 (6.0)	542 (3.9)	940 (8.5)	<0.001⁎⁎⁎
Cerebral infarction (%)	496 (2.0)	286 (2.1)	210 (1.9)	0.4
CKD (%)	5733 (23)	3021 (22)	2712 (25)	<0.001⁎⁎⁎
COPD (%)	3013 (12)	1424 (10)	1589 (14)	<0.001⁎⁎⁎
Dementia (%)	1025 (4.1)	236 (1.7)	789 (7.1)	<0.001⁎⁎⁎
Depression (%)	2546 (10)	974 (7.0)	1572 (14)	<0.001⁎⁎⁎
Diabetes (%)	7723 (31)	4128 (30)	3595 (33)	<0.001⁎⁎⁎
Hepatic failure (%)	1663 (6.7)	624 (4.5)	1039 (9.4)	<0.001⁎⁎⁎
Hypertension (%)	9978 (40)	5778 (42)	4200 (38)	<0.001⁎⁎⁎
Hypovolemic (%)	3920 (16)	2091 (15)	1829 (17)	0.002⁎⁎
IHD (%)	8733 (35)	4958 (36)	3775 (34)	0.008⁎⁎
Liver cirrhosis (%)	1490 (6.0)	834 (6.0)	656 (5.9)	0.8
Malignant cancer (%)	3985 (16)	2313 (17)	1672 (15)	<0.001⁎⁎⁎
Respiratory failure (%)	9180 (37)	3486 (25)	5694 (52)	<0.001⁎⁎⁎
APS III	46 [34, 61]	43 [32, 57]	49 [37, 64]	<0.001⁎⁎⁎
LODS	5 [3, 7]	4 [3, 6]	5 [4, 8]	<0.001⁎⁎⁎
SIRS (%)				<0.001⁎⁎⁎
0	244 (1.0)	135 (1.0)	109 (1.0)	
1	2063 (8.3)	1175 (8.5)	888 (8.0)	
2	6823 (27)	3714 (27)	3109 (28)	
3	10,507 (42)	5663 (41)	4844 (44)	
4	5252 (21)	3162 (23)	2090 (19)	
SOFA	5 [3, 8]	5 [3, 7]	6 [4, 8]	<0.001⁎⁎⁎
GCS	15 [13, 15]	15 [14, 15]	14 [13, 15]	<0.001⁎⁎⁎
ACEI (%)	3018 (12)	2546 (18)	472 (4.3)	<0.001⁎⁎⁎
Antibiotic (%)	21,105 (85)	13,786 (100)	7319 (66)	<0.001⁎⁎⁎
Vasoactive (%)	9192 (37)	5569 (40)	3623 (33)	<0.001⁎⁎⁎
Sedative (%)	6227 (25)	3617 (26)	2610 (24)	<0.001⁎⁎⁎
Creatinine (mg/dL)	1.00 [0.80, 1.60]	1.00 [0.80, 1.50]	1.10 [0.80, 1.70]	<0.001⁎⁎⁎
Glucose (mg/dL)	127 [103, 165]	124 [102, 160]	131 [105, 172]	<0.001⁎⁎⁎
Hematocrit (%)	33 [28, 38]	32 [28, 37]	33 [28, 38]	<0.001⁎⁎⁎
Hemoglobin (g/dL)	10.70 [9.10, 12.40]	10.70 [9.10, 12.30]	10.80 [9.10, 12.50]	0.018*
INR	1.30 [1.10, 1.50]	1.30 [1.10, 1.50]	1.30 [1.10, 1.60]	0.064
Lactate (%)				<0.001⁎⁎⁎
> 2	7503 (30)	3938 (28)	3565 (32)	
≤ 2	10,500 (42)	6557 (47)	3943 (36)	
Missing	6886 (28)	3354 (24)	3532 (32)	
Platelets (10^9/L)	190 [134, 256]	190 [134, 258]	190 [135, 255]	0.3
PT (*sec*)	14 [13,17]	14 [13, 17]	14 [12, 17]	<0.001⁎⁎⁎
APTT (*sec*)	31 [27, 37]	31 [27, 37]	31 [27, 37]	0.009⁎⁎
Urea nitrogen (mg/dL)	21 [14, 36]	20 [14, 33]	23 [15, 38]	<0.001⁎⁎⁎
WBC (10^9/L)	11 [8, 16]	11 [7, 15]	11 [8, 16]	<0.001⁎⁎⁎
Heart rate (beats/min)	88 [76, 103]	87 [76, 101]	90 [77, 105]	<0.001⁎⁎⁎
Non invasive blood pressure diastolic (mmHg)	66 [56, 78]	64 [55, 76]	68 [58, 81]	<0.001⁎⁎⁎
Non invasive blood pressure mean (mmHg)	79 [69, 92]	77 [68, 89]	82 [71, 95]	<0.001⁎⁎⁎
Non invasive blood pressure systolic (mmHg)	119 [104, 136]	117 [103, 135]	120 [104, 138]	<0.001⁎⁎⁎
Respiratory rate (beats/min)	19 [15, 23]	18 [15, 22]	20 [16, 24]	<0.001⁎⁎⁎
SPO_2_ (%)	98.0 [95.0, 100.0]	98.0 [95.0, 100.0]	98.0 [95.0, 100.0]	<0.001⁎⁎⁎
Hospital time (days)	8 [5, 15]	6 [4, 10]	12 [7, 20]	<0.001⁎⁎⁎
ePWV (m/s)	9.70 [7.72, 12.06]	9.44 [7.54, 11.77]	10.00 [8.00, 12.40]	<0.001⁎⁎⁎
ePWV_q (%)				<0.001⁎⁎⁎
Q1	6223 (25)	3811 (28)	2412 (22)	
Q2	6237 (25)	3562 (26)	2675 (24)	
Q3	6223 (25)	3344 (24)	2879 (26)	
Q4	6206 (25)	3132 (23)	3074 (28)	

ARF, Acute Renal Failure; CKD, Chronic Kidney Diseases; COPD, Chronic Obstructive Pulmonary Disease; IHD, Ischemic Heart Disease; APS III, Acute Physiology Score III; LODS, Logistic Organ Dysfunction Score; SIRS, Systemic Inflamma, ry, Response Syndromecells; SPOpsis-related Organ Failure Assessment; GCS, Glasgow Coma Scale; ACEI, Angiotensin-Converting Enzyme Inhibitors; INR, International Normalized Ratio; PT, Prothrombin Time; APTT, Activated Partial Thromboplastin Time; WBC, White Blood Cells; SPO_2_, Pulse Oxime-try-derived Oxygen Saturation.

a Median [Q1, Q3]; n (%).

b Wilcoxon rank sum test; Pearson's Chi-Squared test.

c *p < 0.05;.

⁎⁎ p < 0.01;.

⁎⁎⁎ p < 0.001.

Table S1 presents the analysis based on quartiles of ePWV. The incidence of delirium increased progressively with higher ePWV quartiles, rising from 39% in Q1 to 50% in Q4. Meanwhile, the median age also increased from 50 to 85 (all p < 0.001). In Q4, comorbidities such as ARF, AF, cardiac failure, CH, CKD, COPD, diabetes, hypertension, IHD, and RF were more prevalent (all p < 0.001). Q4 also exhibited more severe clinical conditions, reflected by higher APS III and SIRS scores and lower GCS scores (all p < 0.001). Laboratory findings in the higher ePWV quartiles showed elevated levels of creatinine, glucose, and platelets, while HGB did not differ significantly (p = 0.11). Differences in ePWV were significant in all quartiles (all p < 0.001).

### KM survival analysis

**Fig. 2:**
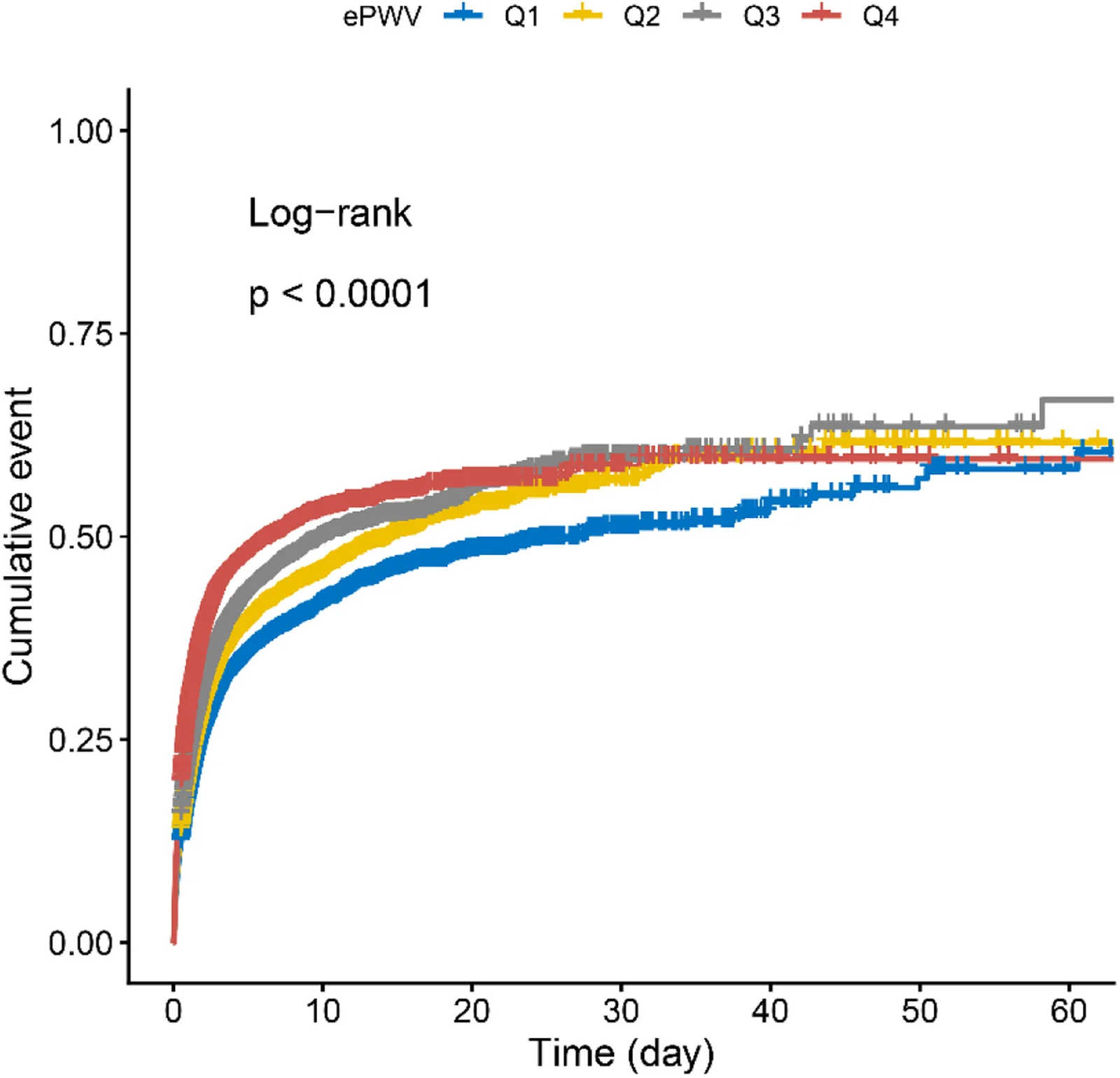
Kaplan-Meier curves showing the occurrence of delirium in different quartiles of ePWV.

KM curve analysis demonstrated significant differences in the delirium risk among patients with different ePWV levels (log-rank p < 0.0001). The lowest incidence of delirium was observed in Q1, whereas the highest incidence was observed in Q4 (Fig. 2).

### Feature selection results

**Fig. 3:**
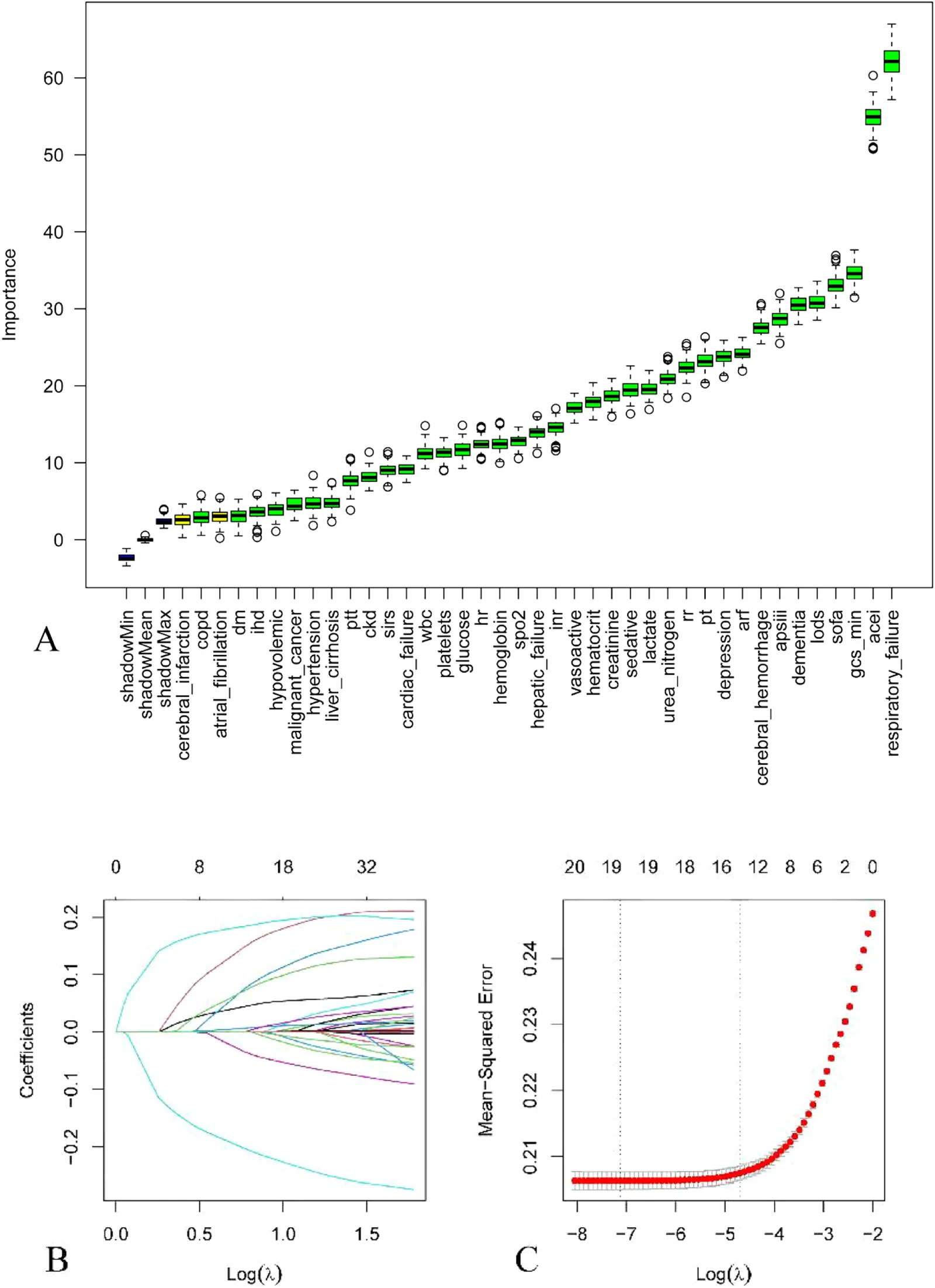
Feature selection results. (A) Boruta analysis results; (B) LASSO regression path diagram; (C) Cross-validation results of LASSO regression.

Feature selection using a combined analytical approach identified key variables associated with delirium. Boruta analysis identified 37 important variables (Fig. 3A), while LASSO regression (λ₁se = 0.0128) retained 25 variables with non-zero coefficients (Fig. 3B). The intersection of these two methods yielded 24-key variables: Acute Respiratory Failure (ARF), cardiac failure, CH, COPD, dementia, depression, HF, hypertension, hypovolemia, liver cirrhosis, malignant cancer, RF, LODS score, SIRS score, SOFA score, ACEI, vasoactive drugs, glucose, HCT, lactate, APTT, heart rate, RR, and sedative use (Fig. 3C).

### Association of ePWV with delirium risk in sepsis patients

Cox proportional hazards regression analysis revealed a significant and independent positive relation of ePWV to delirium risk in the sepsis cohort. When ePWV was a continuous variable, the delirium risk increased significantly with higher ePWV levels. This association remained robust after adjustment for all confounding factors (HR = 1.018, 95% CI: 1.008‒1.027, p < 0.001). When ePWV was analyzed as quartiles, the delirium risk rose stepwise with increasing ePWV levels. Compared with Q1, Q4 had an 19% higher delirium risk (HR = 1.193, 95% CI: 1.105‒1.287, p < 0.001). Our trend test showed consistent results (fully adjusted HR = 1.135, 95% CI: 1.074‒1.199, p < 0.001). Results are provided in Table 2.

**Table 2:** Association between ePWV and delirium in patients with sepsis.

Categories	Model 1			Model 2			Model 3		
	**HR**	**95% CI**	**p-value**	**HR**	**95% CI**	**p-value**	**HR**	**95% CI**	**p-value**
**COX**									
ePWV (continuous)	1.054	1.047, 1.061	<0.001	1.072	1.063, 1.081	<0.001	1.018	1.008, 1.027	<0.001
log(ePWV)	1.658	1.553, 1.770	<0.001	2.051	1.875, 2.244	<0.001	1.221	1.111, 1.343	<0.001
ePWV (quartiles)									
Q1	Ref.	Ref.		Ref.	Ref.		Ref.	Ref.	
Q2	1.141	1.080, 1.205	<0.001	1.241	1.166, 1.321	<0.001	1.145	1.075, 1.219	<0.001
Q3	1.261	1.195, 1.331	<0.001	1.426	1.329, 1.530	<0.001	1.188	1.105, 1.277	<0.001
Q4	1.466	1.390, 1.547	<0.001	1.687	1.569, 1.814	<0.001	1.193	1.105, 1.287	<0.001
p of trend	1.322	1.273, 1.373	<0.001	1.465	1.390, 1.544	<0.001	1.135	1.074, 1.199	<0.001
**Fine-Gray**									
ePWV (continuous)	1.081	1.034, 1.131	<0.001	1.164	1.507, 0.899	<0.001	1.109	1.284, 0.957	<0.001
log(ePWV)	2.032	1.282, 3.221	0.003	4.241	5.662, 3.176	0.002	2.832	3.345, 2.398	0.001
ePWV (quartiles)									
Q1	Ref.	Ref.		Ref.	Ref.		Ref.	Ref.	
Q2	1.035	0.364, 2.945	0.83	1.258	0.199, 7.956	0.26	1.302	0.283, 5.988	0.19
Q3	1.07	0.364, 2.945	0.75	1.502	0.199, 7.956	0.17	1.487	0.283, 5.988	0.15
Q4	1.705	0.364, 2.945	0.002	2.563	0.199, 7.956	0.003	2.178	0.283, 5.988	0.002

Model 1: Unadjusted; Model 2: Adjusted for age, gender and race; Model 3: Adjusted for age, gender, race, ARF, Cardiac failure, Cerebral hemorrhage, COPD, Dementia, Depression, Hepatic failure, Hypertension, Hypovolemic, Liver cirrhosis, Malignant cancer, RF, LODS, SIRS, SOFA, ACEI, Vasoactive, Glucose, HCT, Lactate, APTT, Heart rate, Respiratory rate, Sedative.

HR, Hazard Ratio; CI, Confidence Interval; ARF, Acute Renal Failure; CPOD, Chronic Obstructive Pulmonary Disease; RF, Respiratory Failure; HCT, Hematocrit; APTT, Activated Partial Thromboplastin Time; SOFA, Sepsis-related Organ Failure Assessment; SIRS, Systemic Inflamma ry Response Syndrome; LODS, Logistic Organ Dysfunction Score; ACEI, Angiotensin-Converting Enzyme Inhibitors.

To further evaluate the incremental predictive value of ePWV, we compared the discriminative performance of two models based on the variables in Model 3, with and without the inclusion of ePWV. ROC analysis demonstrated that the baseline model without ePWV yielded an Area Under the Curve (AUC) of 0.703 (95% CI: 0.697‒0.710), whereas the inclusion of ePWV increased the AUC to 0.707 (95% CI: 0.701‒0.714). DeLong’s test confirmed that the model incorporating ePWV exhibited significantly superior discriminative performance compared with the baseline model (p < 0.001) (Fig. S1).

To verify the robustness of the primary analysis, a Fine-Gray subdistribution hazard model was employed, with ICU mortality treated as a competing risk event, to reassess the association between ePWV and delirium risk. The Fine-Gray model demonstrated that, after adjustment for the same covariates, each one-unit increase in ePWV was associated with a Subdistribution Hazard Ratio (SHR) for delirium of 1.109 (95% CI: 0.957‒1.284), while the highest Quartile group (Q4) exhibited an SHR of 2.178 (95% CI: 0.283‒5.988) compared with the lowest Quartile group (Q1). These findings were consistent in direction with the effect estimates derived from the primary Cox regression analysis, indicating that the presence of competing risks did not materially alter the conclusion that ePWV was independently associated with delirium risk in ICU patients with sepsis. Results are presented in Table 2.

**Fig. 4:**
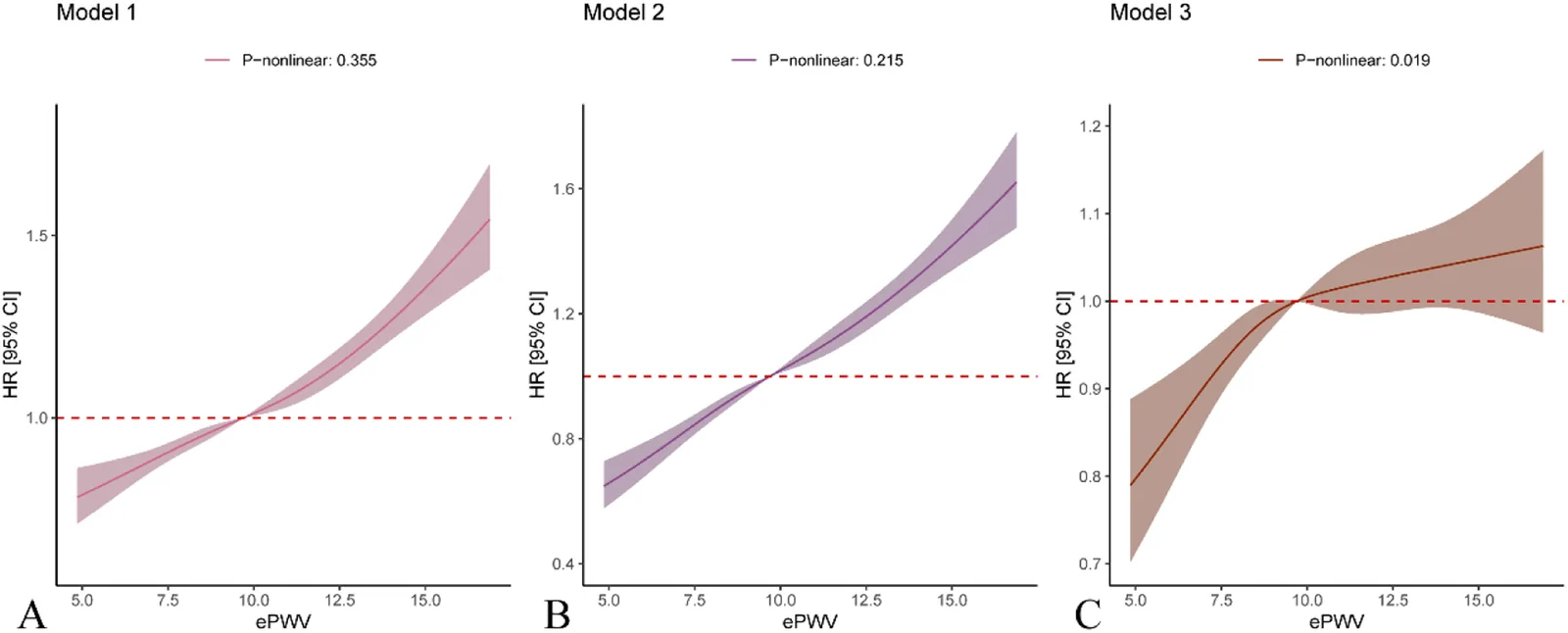
Restricted cubic spline analysis depicted the relationship between ePWV and the risk of delirium. (A) Model 1; (B) Model 2; (C) Model 3. The solid line represents the Hazard Ratio (HR), and the shaded area indicates the 95% Confidence Interval.

RCS analysis indicated that the delirium risk increased approximately linearly with rising ePWV in Model 1 (p for nonlinear = 0.355) and Model 2 (p for nonlinear = 0.215). Nevertheless, in Model 3, a significant nonlinear relation of ePWV to delirium risk was noted (p for nonlinear = 0.019) (Fig. 4).

**Fig. 5:**
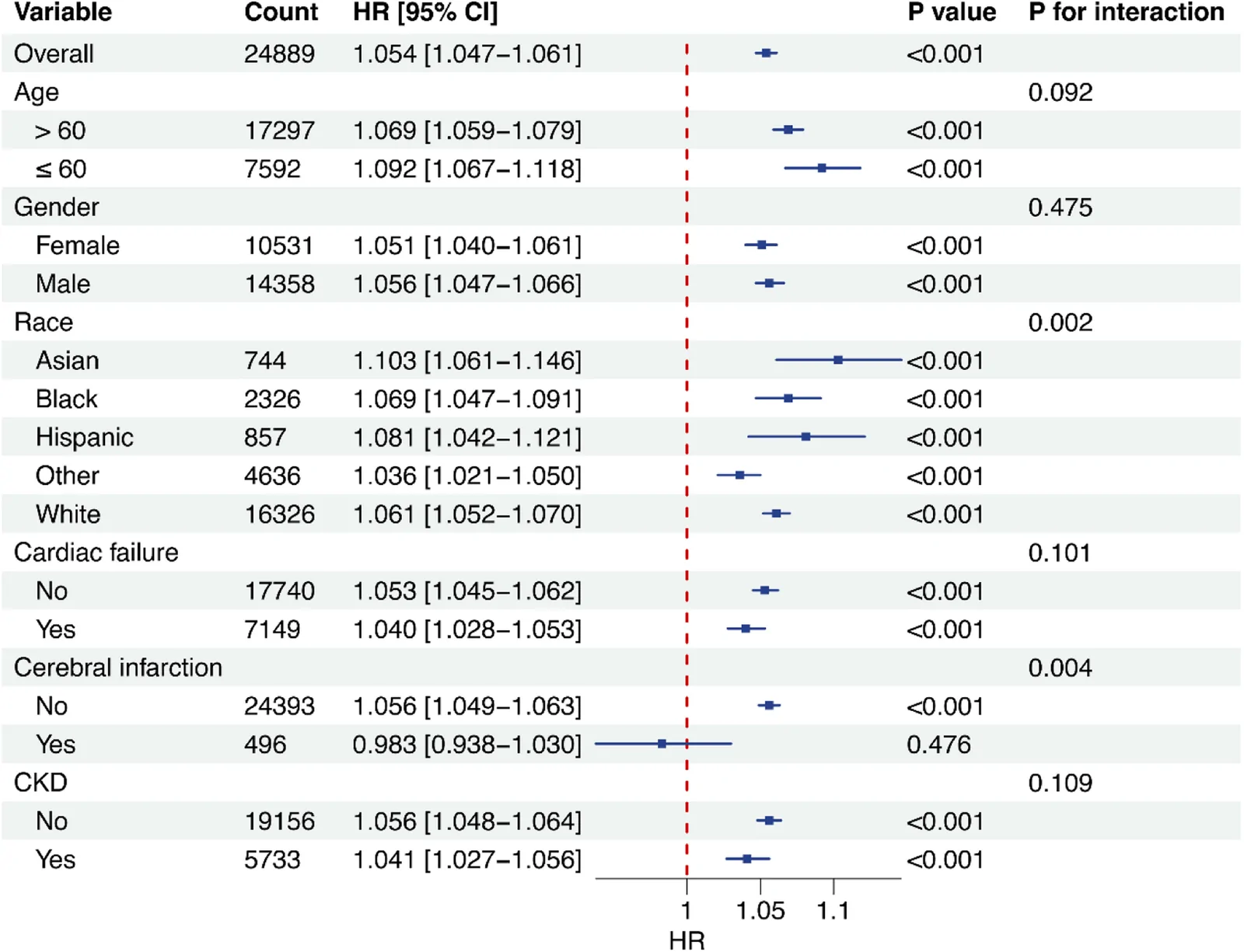
Subgroup analysis of ePWV and the risk of delirium. CKD, Chronic Kidney Disease; CI, Confidence Interval; HR, Hazard Ratio.

Subgroup analyses by age, sex, ethnicity, cardiac failure, cerebral infarction, and CKD showed a significant relation of ePWV to delirium risk across subgroups except for cerebral infarction patients (all p < 0.001). In addition, interactions were significant between ePWV and ethnicity (p = 0.002) as well as cerebral infarction (p = 0.004) (Fig. 5).

### Development and validation of predictive models

Detailed model parameters are provided in Table S2.

**Fig. 6:**
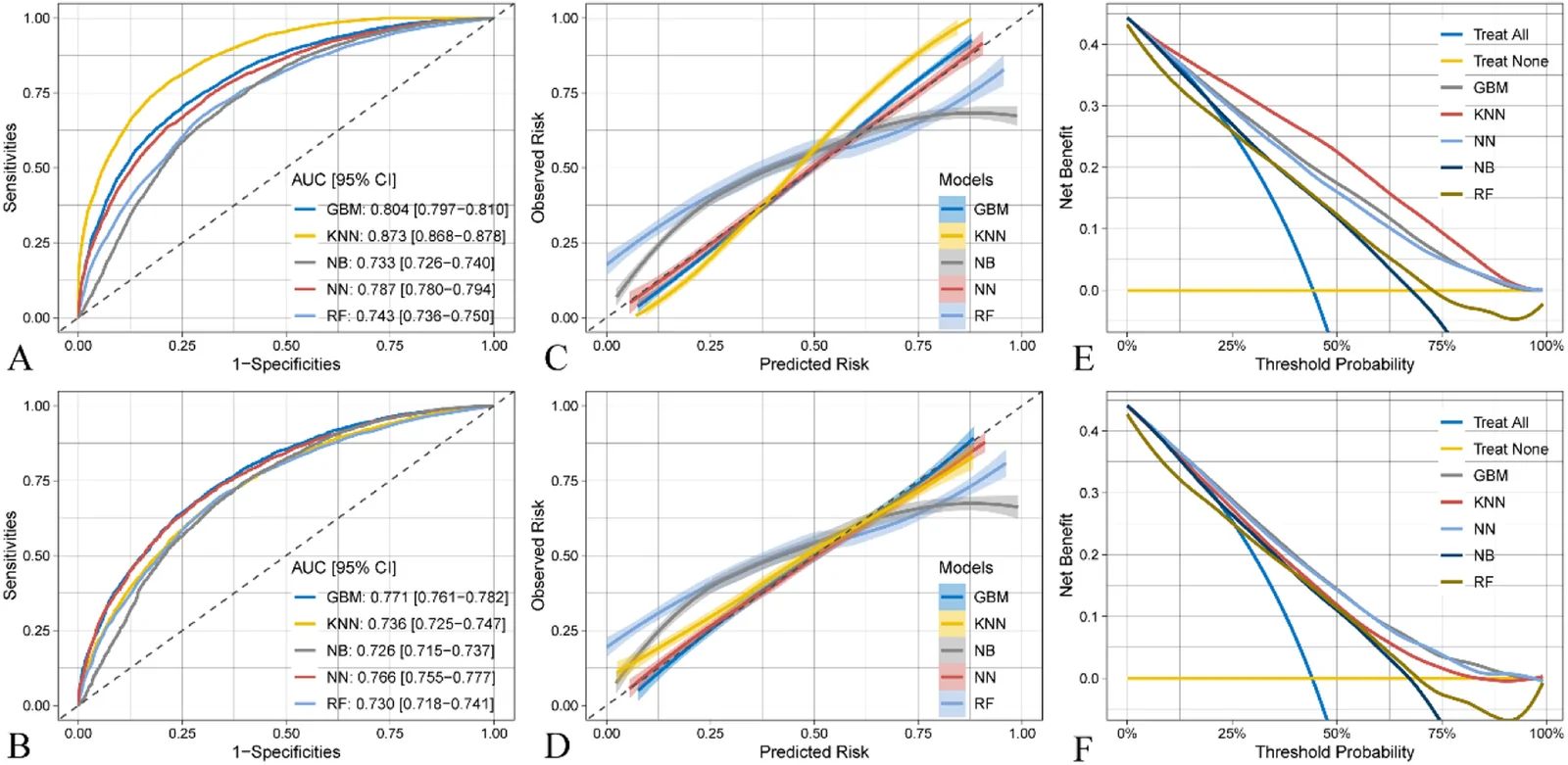
Risk of delirium in patients with sepsis in the training set and validation set. (A) ROC curve analysis of the training cohort; (B) ROC curve analysis of the validation cohort; (C) Calibration curve analysis of the training cohort; (D) Calibration curve analysis of the validation cohort; (E) Decision curve analysis of the training cohort; (F) Decision curve analysis of the validation cohort.

ROC curve analysis demonstrated that all five models: GBM, KNN, NB, NN, and RF, exhibited good predictive performance. In the training cohort, the AUC values were: GBM, 0.804 (95% CI: 0.797‒0.810); KNN, 0.873 (95% CI: 0.868‒0.878); NB, 0.733 (95% CI: 0.726‒0.740); NN, 0.787 (95% CI: 0.780‒0.794); and RF, 0.743 (95% CI: 0.736‒0.750). In the validation cohort, the AUC values of the five models were: GBM, 0.771 (95% CI: 0.761‒0.782); KNN, 0.736 (95% CI: 0.725‒0.747); NB, 0.726 (95% CI: 0.715‒0.737); NN, 0.766 (95% CI: 0.755‒0.777); and RF, 0.730 (95% CI: 0.718–0.741) (Fig. 6A‒B).

All models showed good calibration, with strong agreement between predicted and observed risks, with insignificant overestimation or underestimation of delirium risk (Fig. 6C‒D).

Decision curve analysis indicated a favorable clinical net benefit of all models. Among them, KNN yielded the greatest net benefit in the training group, whereas NN and GBM performed best in the validation cohort (Fig. 6E‒F).

Performance metrics, including sensitivity, specificity, balanced accuracy, and F1 score, were further evaluated. In the training cohort, the KNN model achieved the highest balanced accuracy and F1 score (0.781 and 0.800, respectively), whereas in the validation cohort, the GBM model demonstrated the highest balanced accuracy and F1 score (0.698 and 0.719, respectively). Detailed results are presented in Table S3.

To further compare model performance, IDI and NRI were calculated for pairwise model comparisons in both cohorts. An NRI > 0 indicates improved classification accuracy, while an IDI > 0 reflects enhanced discrimination ability.

The results further showed that, in the validation cohort, the KNN model achieved significantly superior NRI and IDI compared with all competing models (all p < 0.001). Relative to the other models, the KNN model demonstrated markedly better classification accuracy and discriminative ability, indicating superior predictive performance within this dataset. Accordingly, a Shiny-based application implementing the KNN model was developed and is available at https://epwvdeliriumsepsismodel.shinyapps.io/shiny/ (Table 3).

**Table 3:** Analysis of NRI and IDI for the training set and validation set of the model.

	Model	NRI		IDI	
		HR (95% CI)	p-value	HR (95% CI)	p-value
**Training Set**	KNN vs. RF	0.1421 (0.113, 0.1713)	p < 0.001	−0.044 (−0.0506, −0.0373)	p < 0.001
	KNN vs. NB	−0.1203 (−0.15, −0.0907)	p < 0.001	−0.0722 (−0.0786, −0.0658)	p < 0.001
	KNN vs. NN	−0.4522 (−0.4811, −0.4233)	p < 0.001	−0.0722 (−0.0762, −0.0683)	p < 0.001
	KNN vs. GBM	−0.4613 (−0.4901, −0.4324)	p < 0.001	−0.0697 (−0.0736, −0.0659)	p < 0.001
**Validation Set**	KNN vs. RF	0.3666 (0.3224, 0.4109)	p < 0.001	0.0599 (0.0497, 0.0701)	p < 0.001
	KNN vs. NB	0.2723 (0.227, 0.3176)	p < 0.001	0.0384 (0.0286, 0.0483)	p < 0.001
	KNN vs. NN	0.1211 (0.0764, 0.1659)	p < 0.001	0.0284 (0.0219, 0.0349)	p < 0.001
	KNN vs. GBM	0.0693 (0.0243, 0.1142)	p = 0.003	0.0211 (0.0146, 0.0276)	p < 0.001

NRI, Net Reclassiflcation Improvement; IDI, Integrated Discrimination Improvement; HR, Hazard Ratio; CI, Confidence Interval.

**Fig. 7:**
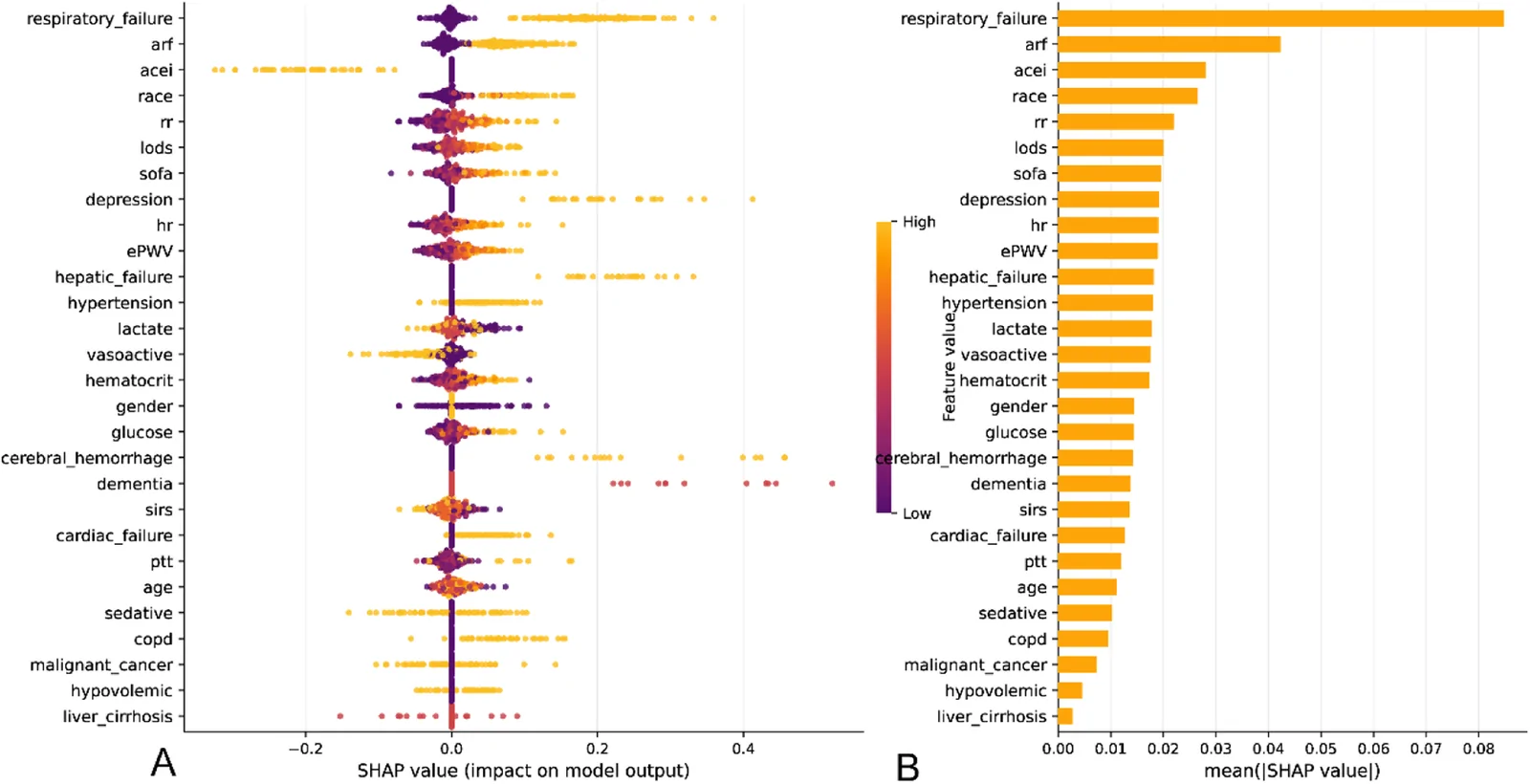
SHAP analysis. (A) SHAP summary plot of the KNN model. (B) Feature importance ranking chart of the KNN model. SOFA, Sepsis-related Organ Failure Assessment; ARF, Acute Renal Failure; ACEI, Angiotensin-Converting Enzyme Inhibitors; LODS, Logistic Organ Dysfunction Score; RR, Respiratory Rate; ePWV, Estimated Pulse Wave Velocity; SIRS, Systemic Inflammatory Response Syndrome; APTT, activated partial thromboplastin time; COPD, chronic obstructive pulmonary disease.

The KNN model was further interpreted using SHAP. Fig. 7B illustrates the hierarchical importance of features in the KNN model, where all variables had mean absolute SHAP values greater than zero. Respiratory Failure (RF) emerged as the most important feature, followed by ARF and ACEI.The SHAP summary plot (Fig. 7A) depicts each feature’s association with the model. Each point represents a feature’s SHAP value for an individual observation, with features listed along the vertical axis and SHAP value magnitude along the horizontal axis, reflecting their impact on the model output. Point color corresponds to the actual feature value, ranging from purple for low values to yellow for high values. Taking the Respiratory Failure (RF) feature as an example, the dense cluster of yellow points on the right side of the zero baseline indicated that presence of RF (high feature value) was associated with an increased risk of delirium in patients with sepsis, whereas the purple points on the left side suggested that absence of RF (low feature value) was associated with a reduced risk of delirium.

## Discussion

Leveraging the MIMIC-IV 3.1, our large-scale retrospective cohort study systematically first unraveled the association of ePWV with delirium risk in the ICU sepsis population, and further constructed and validated a clinical predictive model incorporating ePWV. The incidence of delirium in ICU sepsis patients was 44.4%, and a significant positive association of ePWV with delirium risk was noted, with Q4 exhibiting an 19% higher risk relative to Q1, and a nonlinear relationship was identified between the two. Subgroup analyses confirmed that this association remained significant across most populations except for those with cerebral infarction, with significant interactions observed for ethnicity and cerebral infarction. Five ML models were constructed and compared, among which the KNN model exhibited the best discriminative performance. SHAP analysis further demonstrated that ePWV exhibited a strong association in model prediction. These findings provide a novel noninvasive biomarker reference for assessing delirium risk in ICU patients with sepsis and may support clinical risk stratification.

AS is characterized by reduced arterial elasticity and is commonly related to aging, atherosclerosis, hypertension, and other cardiovascular and cerebrovascular diseases.^23^ PWV, as a recognized AS marker, predicts cardiovascular events by measuring the speed at which pressure waves propagate through the arteries.^24^^,^^25^ ePWV derived from age and BP is a practical and reliable alternative to direct PWV measurement and has strong agreement with direct methods.^11^ Its simplicity and accessibility make it particularly suitable for both research and clinical practice. In the heart failure population with preserved ejection fraction, Xue et al. demonstrated the relation of higher ePWV to elevated all-cause and cardiovascular mortality risks, with the highest quartile (Epwv ≥12.806 m/s) exhibiting nearly double the mortality risk.^26^ Zhang et al. further reported that ePWV is possibly associated with mortality in the acute ischemic stroke population, with elevated ePWV independently related to increased mortality risk.^27^ Similarly, Wei et al. found a strong association between high ePWV and increased 28-day mortality in patients with acute kidney injury combined with congestive heart failure (HR = 1.87, 95% CI: 1.68‒2.08, p < 0.001), with a linear rise in mortality risk as ePWV increased.^28^ Moreover, Liu et al. reported the significantly nonlinear relation of higher ePWV to short-term and long-term mortality in the critically ill sepsis population.^29^ Our findings align with these prior findings, further reflecting the promising utility of ePWV in delirium risk assessment among sepsis patients and expanding its application in critical care settings.

Increased AS (reflected by elevated ePWV) may be associated with the development of sepsis-associated delirium through multiple mechanisms. First, reduced arterial compliance may impair cerebral autoregulation. In the context of sepsis-related microcirculatory dysfunction, this can further decrease cerebral perfusion, leading to cerebral ischemia and hypoxia, disrupting neuronal metabolism, and ultimately resulting in clinical manifestations of delirium such as altered consciousness and impaired attention.^30^^,^^31^ Second, increased AS reflects endothelial dysfunction. Damaged endothelium releases inflammatory mediators, which may amplify the systemic inflammatory response in sepsis, activate neuroinflammatory pathways, promote microglial activation, and increase blood-brain barrier permeability, ultimately leading to acute brain dysfunction. Finally, elevated ePWV is more commonly observed in elderly patients and those with hypertension or CKD, populations that often have pre-existing impairments in brain structure and cognitive reserve. Under the stress of sepsis, these individuals are more susceptible to developing delirium.^32^ It should also be noted that patients who develop delirium early may experience hemodynamic alterations secondary to pain, agitation, or delirium itself.^33^

Our subgroup analyses showed a consistent relation of ePWV to delirium risk in most subgroups, suggesting that the relation of ePWV to delirium risk is robust under various clinical conditions. However, significant interactions were observed in certain subgroups, indicating that specific patient characteristics may modify the effect of ePWV on delirium risk. Notably, the relation of ePWV to delirium risk was not significant in patients with a history of cerebral infarction. Several explanations may account for this finding. First, prior cerebral infarction itself may result in structural brain damage and reduced cognitive reserve, leading to an inherently high baseline delirium risk, thereby attenuating the relative association with AS, which may act as a “secondary associated variable”.^34^ Second, patients with cerebral infarction often receive standardized secondary prevention therapies (e.g., BP and lipid control), which may partially mitigate the adverse effects of AS. Third, the pathophysiological mechanisms of post-stroke delirium may involve focal neurological deficits and lesion-specific effects, thereby weakening the relation to systemic AS.^35^^,^^36^ Moreover, the interaction between ePWV and ethnicity was significant (p = 0.002), with a seemingly stronger predictive effect of ePWV among non-White populations. This may be attributed to differences in vascular physiology, baseline disease profiles, and genetic backgrounds across ethnic groups.

A key strength of this study lies in the application of ML techniques to identify important predictive factors and construct predictive models. ML models are capable of capturing nonlinear associations and interaction effects, and demonstrated favorable predictive performance in the present study. Given that traditional ML models are often regarded as “black boxes” with limited transparency in their decision-making processes, SHAP analysis was employed to enhance the interpretability of the KNN model. Through SHAP summary plots and feature importance visualizations, the direction and magnitude of the effects of individual feature values on delirium risk were clearly illustrated, thereby substantially improving model transparency. To further enhance clinical accessibility, an interactive web-based risk prediction tool based on the KNN model was developed as a Shiny application (https://epwvdeliriumsepsismodel.shinyapps.io/shiny/). This tool integrates the 24 key predictive variables identified by the model. Clinicians can obtain individualized probabilities and risk stratification categories (low-, intermediate-, or high-risk) for delirium in real time by entering patient information, including age, ePWV, comorbidities (e.g., RF and ARF), laboratory indicators (e.g., SOFA score and lactate), and medication use (e.g., ACEI administration). The output format is intuitive and user-friendly, requiring no specialized programming expertise, thereby effectively overcoming the technical limitations of SHAP-value presentation and facilitating the translation from “model output” to “clinical decision support”.

SHAP analysis identified the strong associations of RF, ARF, and ACEI with sepsis-associated delirium within the KNN model. RF was the most important feature, which may be explained by its direct association with cerebral hypoxia. Inadequate oxygenation, impaired cerebral microcirculatory perfusion, and neuronal metabolic dysfunction during RF are associated with sepsis-associated delirium.^37^ ARF, as the second most important feature, reflects the critical role of renal dysfunction in delirium pathogenesis. Acute kidney injury leads to accumulation of uremic toxins, electrolyte imbalances, and fluid overload, all of which can disrupt neuronal function and increase delirium susceptibility.^38^ ACEI ranked third among the predictive features. By inhibiting angiotensin-converting enzyme and reducing angiotensin II production, ACEIs may improve cerebral blood flow autoregulation while attenuating oxidative stress and neuroinflammatory responses.^39^ Together with ePWV, these three core features form a multidimensional risk assessment framework composed of routinely obtainable clinical indicators with strong practical applicability.

Our study preliminarily demonstrated an independent association between ePWV and delirium risk in ICU patients with sepsis and established a risk stratification model with favorable discriminative performance. However, several limitations should be acknowledged. First, all data came from MIMIC-IV, a single-center database, which possibly limits the generalizability of our findings owing to population homogeneity. Second, in a retrospective cohort study, the causal relation of ePWV to delirium risk cannot be entirely established, and only associations can be inferred. Finally, due to limitations in available variables within the database, certain potentially relevant factors, such as genetic data, echocardiographic findings, neuroimaging, and lifestyle variables (e.g., smoking and physical activity), were not included, which may have influenced the results. Future studies should include large-scale, multicenter cohorts to externally validate the model and prospective designs to confirm causal relationships. Additionally, integrating multimodal data (imaging and molecular markers) possibly enables more comprehensive analyses.

## Conclusion

ePWV was independently associated with delirium risk in ICU patients with sepsis, and the KNN model integrating ePWV exhibited good predictive performance and clinical interpretability, thereby facilitating clinical risk stratification and the early identification of patients at high risk of delirium. Future prospective multicenter studies are warranted to clarify the causal relationship and further elucidate the potential mechanistic links between AS and delirium.

## Data availability

All data generated or analysed during this study are included in this published article and its supplementary information files.

## Author’s contribution

All authors contributed to the study conception and design. Lihong Dong: Data curation, Conceptualization, Methodology, Writing-original draft preparation. Xixiang Yang: Visualization, Investigation, Formal analysis, Software. Xiaolin Zhu: Supervision. Hong Wang: Validation, Project administration. Xiaoyan Wang: Writing-reviewing and editing. All authors commented on previous versions of the manuscript. All authors read and approved the final manuscript, and all authors commented on previous versions of the manuscript. All authors read and approved the final manuscript.

## Funding

This research did not receive any specific grant from funding agencies in the public, commercial, or not-for-profit sectors.

## Conflicts of interest

The authors declare no conflicts of interest.

## Appendix Supplementary materials

Image, application 1
