# Supplementary material for: Association between ePWV and delirium risk in icu sepsis patients: a retrospective analysis based on the MIMIC database and development of a predictive model
Source: Clinics (Sao Paulo). 2026 Sep 5;81:101106. doi: 10.1016/j.clinsp.2026.101106 (PMC13571930; doi:10.1016/j.clinsp.2026.101106)
Supplement: Supplementary file 1 [file mmc1.docx]

**CLINICS-D-26-00728**

**Supplementary Materials**

**Table S1** Baseline characteristics of the study population based on the quartile grouping of ePWV.

| **Characteristic** | **Overall**  **(n = 24,889)^a^** | **Q1**  **(n = 6,223)^a^** | **Q2**  **(n = 6,237)^a^** | **Q3**  **(n = 6,223)^a^** | **Q4**  **(n = 6,206)^a^** | **p-value^b,c^** |
| --- | --- | --- | --- | --- | --- | --- |
| Delirium (%) | 11,040 (44) | 2,412 (39) | 2,675 (43) | 2,879 (46) | 3,074 (50) | <0.001^***^ |
| Follow-up time (delirium) (days) | 4.0 [1.1, 7.4] | 4.3 [1.5, 8.0] | 4.2 [1.3, 7.4] | 4.0 [1.1, 7.5] | 3.0 [0.7, 6.8] | <0.001^***^ |
| Age (years) | 68 [57, 79] | 50 [38, 58] | 64 [58, 69] | 74 [68, 78] | 85 [81, 89] | <0.001^***^ |
| Gender (%) |  |  |  |  |  | <0.001^***^ |
| Female | 10,531 (42) | 2,496 (40) | 2,402 (39) | 2,555 (41) | 3,078 (50) |  |
| Male | 14,358 (58) | 3,727 (60) | 3,835 (61) | 3,668 (59) | 3,128 (50) |  |
| Race (%) |  |  |  |  |  | <0.001^***^ |
| Asian | 744 (3.0) | 203 (3.3) | 170 (2.7) | 168 (2.7) | 203 (3.3) |  |
| Black | 2,326 (9.3) | 558 (9.0) | 578 (9.3) | 622 (10.0) | 568 (9.2) |  |
| Hispanic | 857 (3.4) | 333 (5.4) | 221 (3.5) | 168 (2.7) | 135 (2.2) |  |
| Other | 4,636 (19) | 1,369 (22) | 1,168 (19) | 1,097 (18) | 1,002 (16) |  |
| White | 16,326 (66) | 3,760 (60) | 4,100 (66) | 4,168 (67) | 4,298 (69) |  |
| ARF (%) | 10,320 (41) | 2,166 (35) | 2,409 (39) | 2,747 (44) | 2,998 (48) | <0.001^***^ |
| Atrial fibrillation (%) | 5,779 (23) | 570 (9.2) | 1,240 (20) | 1,733 (28) | 2,236 (36) | <0.001^***^ |
| Cardiac failure (%) | 7,149 (29) | 861 (14) | 1,452 (23) | 2,109 (34) | 2,727 (44) | <0.001^***^ |
| Cerebral hemorrhage (%) | 1,482 (6.0) | 260 (4.2) | 370 (5.9) | 401 (6.4) | 451 (7.3) | <0.001^***^ |
| Cerebral infarction (%) | 496 (2.0) | 109 (1.8) | 109 (1.7) | 126 (2.0) | 152 (2.4) | 0.015^*^ |
| CKD (%) | 5,733 (23) | 752 (12) | 1,160 (19) | 1,700 (27) | 2,121 (34) | <0.001^***^ |
| COPD (%) | 3,013 (12) | 340 (5.5) | 777 (12) | 980 (16) | 916 (15 ) | <0.001^***^ |
| Dementia (%) | 1,025 (4.1) | 22 (0.4) | 71 (1.1) | 224 (3.6) | 708 (11) | <0.001^***^ |
| Depression (%) | 2,546 (10) | 704 (11) | 717 (11) | 620 (10.0) | 505 (8.1) | <0.001^***^ |
| Diabetes (%) | 7,723 (31) | 1,396 (22) | 2,091 (34) | 2,269 (36) | 1,967 (32) | <0.001^***^ |
| Hepatic failure (%) | 1,663 (6.7) | 569 (9.1) | 457 (7.3) | 381 (6.1) | 256 (4.1) | <0.001^***^ |
| Hypertension (%) | 9,978 (40) | 1,800 (29) | 2,765 (44) | 2,791 (45) | 2,622 (42) | <0.001^***^ |
| Hypovolemic (%) | 3,920 (16) | 943 (15) | 1,036 (17) | 1,006 (16) | 935 (15) | 0.043^*^ |
| IHD (%) | 8,733 (35) | 1,215 (20) | 2,151 (34) | 2,587 (42) | 2,780 (45) | <0.001^***^ |
| Liver cirrhosis (%) | 1,490 (6.0) | 553 (8.9) | 506 (8.1) | 318 (5.1) | 113 (1.8) | <0.001^***^ |
| Malignant cancer (%) | 3,985 (16) | 764 (12) | 1,097 (18) | 1,184 (19) | 940 (15) | <0.001^***^ |
| Respiratory failure (%) | 9,180 (37) | 2,044 (33) | 2,206 (35) | 2,444 (39) | 2,486 (40) | <0.001^***^ |
| APS III | 46 [34, 61] | 43 [31, 60] | 44 [32, 59] | 46 [35, 61] | 48 [39, 62] | <0.001^***^ |
| LODS | 5 [3, 7] | 4 [3, 7] | 5 [3, 7] | 5 [3, 7] | 5 [3, 7] | <0.001^***^ |
| SIRS (%) |  |  |  |  |  | <0.001^***^ |
| 0 | 244 (1.0) | 42 (0.7) | 65 (1.0) | 75 (1.2) | 62 (1.0) |  |
| 1 | 2,063 (8.3) | 399 (6.4) | 470 (7.5) | 567 (9.1) | 627 (10) |  |
| 2 | 6,823 (27) | 1,377 (22) | 1,685 (27) | 1,800 (29) | 1,961 (32) |  |
| 3 | 10,507 (42) | 2,709 (44) | 2,686 (43) | 2,586 (42) | 2,526 (41) |  |
| 4 | 5,252 (21) | 1,696 (27) | 1,331 (21) | 1,195 (19) | 1,030 (17) |  |
| SOFA | 5 [3, 8] | 5 [3, 8] | 5 [3, 8] | 5 [3, 7] | 5 [3, 7] | 0.3 |
| GCS | 15 [13, 15] | 15 [14, 15] | 15 [14, 15] | 15 [13, 15] | 14 [12, 15] | <0.001^***^ |
| ACEI (%) | 3,018 (12) | 516 (8.3) | 825 (13) | 880 (14) | 797 (13) | <0.001^***^ |
| Antibiotic (%) | 21,105 (85) | 5,505 (88) | 5,394 (86) | 5,255 (84) | 4,951 (80) | <0.001^***^ |
| Vasoactive (%) | 9,192 (37) | 2,502 (40) | 2,462 (39) | 2,378 (38) | 1,850 (30) | <0.001^***^ |
| Sedative (%) | 6,227 (25) | 2,010 (32) | 1,656 (27) | 1,470 (24) | 1,091 (18) | <0.001^***^ |
| Creatinine (mg/dL) | 1.00 [0.80, 1.60] | 0.90 [0.70, 1.40] | 1.00 [0.70, 1.60] | 1.10 [0.80, 1.70] | 1.20 [0.80, 1.70] | <0.001^***^ |
| Glucose (mg/dL) | 127 [103, 165] | 123 [101, 156] | 127 [104, 168] | 130 [105, 170] | 129 [105, 165] | <0.001^***^ |
| Hematocrit (%) | 33 [28, 38] | 33 [28, 38] | 33 [28, 38] | 33 [28, 38] | 33 [29, 37] | 0.006^**^ |
| Hemoglobin (g/dL) | 10.70 [9.10, 12.40] | 10.70 [9.10, 12.50] | 10.70 [9.10, 12.50] | 10.70 [9.10, 12.40] | 10.70 [9.20, 12.10] | 0.11 |
| INR | 1.30 [1.10, 1.50] | 1.30 [1.10, 1.50] | 1.30 [1.10, 1.50] | 1.30 [1.10, 1.50] | 1.30 [1.10, 1.50] | 0.3 |
| Lactate (%) |  |  |  |  |  | <0.001^***^ |
| > 2 | 7,503 (30) | 2,069 (33) | 1,877 (30) | 1,816 (29) | 1,741 (28) |  |
| ≤ 2 | 10,500 (42) | 2,742 (44) | 2,864 (46) | 2,661 (43) | 2,233 (36) |  |
| Missing | 6,886 (28) | 1,412 (23) | 1,496 (24) | 1,746 (28) | 2,232 (36) |  |
| Platelets (10^9/L) | 190 [134, 256] | 187 [126, 254] | 190 [130, 258] | 191 [137, 257] | 192 [141, 257] | <0.001^***^ |
| PT (sec) | 14 [13, 17] | 14 [13, 17] | 14 [13, 17] | 14 [13, 17] | 14 [13, 17] | 0.026^*^ |
| APTT (sec) | 31 [27, 37] | 31 [27, 37] | 31 [27, 37] | 31 [27, 37] | 31 [27, 37] | 0.6 |
| Urea nitrogen (mg/dL) | 21 [14, 36] | 17 [11, 27] | 20 [14, 33] | 23 [16, 38] | 26 [18, 42] | <0.001^***^ |
| WBC (10^9/L) | 11 [8, 16] | 11 [8, 16] | 11 [8, 16] | 11 [8, 15] | 11 [8, 15] | 0.019^*^ |
| Heart rate (beats/min) | 88 [76, 103] | 91 [79, 107] | 88 [77, 103] | 87 [75, 101] | 87 [75, 102] | <0.001^***^ |
| Non invasive blood pressure diastolic (mmHg) | 66 [56, 78] | 61 [53, 70] | 67 [57, 78] | 67 [56, 81] | 70 [58, 86] | <0.001^***^ |
| Non invasive blood pressure mean (mmHg) | 79 [69, 92] | 72 [65, 82] | 79 [70, 90] | 81 [70, 95] | 86 [74, 101] | <0.001^***^ |
| Non invasive blood pressure systolic (mmHg) | 119 [104, 136] | 108 [97, 121] | 117 [103, 133] | 123 [107, 141] | 129 [112, 147] | <0.001^***^ |
| Respiratory rate (beats/min) | 19 [15, 23] | 18 [15, 23] | 18 [15, 23] | 19 [15, 23] | 20 [16, 24] | <0.001^***^ |
| SPO_2_ (%) | 98.0 [95.0, 100.0] | 99.0 [96.0, 100.0] | 98.0 [95.0, 100.0] | 98.0 [95.0, 100.0] | 97.0 [95.0, 100.0] | <0.001^***^ |
| Hospital time (days) | 8 [5, 15] | 8 [5, 16] | 8 [5, 16] | 8 [5, 15] | 8 [5, 13] | <0.001^***^ |
| ePWV (m/s) | 9.70 [7.72, 12.06] | 6.61 [5.96, 7.19] | 8.71 [8.22, 9.20] | 10.77 [10.23, 11.37] | 13.72 [12.83, 14.79] | <0.001^***^ |

^a^ Median [Q1, Q3]; n (%).

^b^ Pearson's Chi-Squared test; Kruskal-Wallis rank sum test.

^c^ *p < 0.05; **p < 0.01; ***p < 0.001.

ARF, Acute Renal Failure; CKD, Chronic Kidney Diseases; COPD, Chronic Obstructive Pulmonary Disease; IHD, Ischemic Heart Disease; APSIII, Acute Physiology Score III; LODS, Logistic Organ Dysfunction Score; SIRS, Systemic Inflamma, ry Response Syndrome; SOFA, Sepsis-related Organ Failure Assessment; GCS, Glasgow Coma Scale; ACEI, Angiotensin-Converting Enzyme Inhibitors; INR, International Normalized Ratio; PT, Prothrombin Time; APTT, Activated Partial Thromboplastin Time; WBC, White Blood Cells; SPO_2_, Pulse Oxime-try-derived Oxygen Saturation.

**Table S2** The optimal parameters of the machine learning model.

| **Model** | **Parameters** |
| --- | --- |
| RF | Ntree = 94; maxnodes = 18 |
| NN | Size = 7; decay = 1; maxit = 351 |
| NB | Laplace = 3.350064 |
| KNN | k = 30 |
| GBM | trees = 500; interaction depth = 3; shrinkage = 0.06 |

**Table S3** Detailed performance indicators of the 5 models.

|  | **Model** | **Sensitivity** | **Specificity** | **Balanced Accuracy** | **F1** |
| --- | --- | --- | --- | --- | --- |
| **Training Set** | GBM | 0.762 | 0.689 | 0.726 | 0.754 |
|  | KNN | 0.824 | 0.739 | 0.781 | 0.8 |
|  | NB | 0.737 | 0.612 | 0.675 | 0.685 |
|  | NN | 0.768 | 0.653 | 0.711 | 0.726 |
|  | RF | 0.654 | 0.709 | 0.682 | 0.743 |
| **Validation Set** | GBM | 0.752 | 0.643 | 0.698 | 0.719 |
|  | KNN | 0.734 | 0.616 | 0.675 | 0.693 |
|  | NB | 0.748 | 0.597 | 0.673 | 0.67 |
|  | NN | 0.74 | 0.653 | 0.696 | 0.727 |
|  | RF | 0.641 | 0.697 | 0.669 | 0.738 |

**Figure S1** Incremental predictive value of ePWV for model discriminative performance.
